# The impact of a disparity-reduction intervention on outcomes of patients with acute coronary syndrome in the emergency department: a clinical trial

**DOI:** 10.1186/s12939-025-02496-1

**Published:** 2025-05-12

**Authors:** Mehdi Moradinia, Sajad Yarahmadi, Mehdi Birjandi, Mohammad Gholami

**Affiliations:** 1 Student Research Committee, School of Nursing and Midwifery, Lorestan University of Medical Sciences, Khorramabad, Iran; 2 Social Determinants of Health Research Center, School of Nursing and Midwifery, Lorestan University of Medical Sciences, Khorramabad, Iran; 3 Nutritional Health Research Center, School of Health and Nutrition, Lorestan University of Medical Sciences, Khorramabad, Iran

**Keywords:** Disparity, Acute coronary syndrome, Emergency department, Patient navigation, Triage

## Abstract

**Background:**

Patients with acute coronary syndrome (ACS) who belong to marginalized groups often do not receive equitable treatment and care when they are referred to emergency departments (ED), and this can have negative consequences for these patients. Therefore, this study aimed to evaluate the impact of a disparity-reduction intervention on outcomes of patients with ACS in the ED.

**Methods:**

This randomized clinical trial included 264 ACS patients, randomly allocated into intervention (*n* = 132) and control group (*n* = 132). The intervention involved improving the triage process by (1) welcoming nurses and (2) conducting specialized triage. Also, a patient navigation (PN) program was implemented, comprising (1) emergency care comprehensive management, (2) supportive education and counseling, and (3) clinical actions with follow-up care. In the control group, standard triage and routine care were provided. Outcomes assessed included pain intensity, patient opinion of pain management, illness perception, threat perception, and short and long-term outcomes.

**Results:**

The results showed that after the intervention, pain intensity and threat perception decreased significantly in the intervention group compared to the control group (*P* < 0.001). Moreover, the opinion of pain management—assessed only post-intervention—was significantly more favorable in the intervention group than in the control group (*P* < 0.001). Illness perception scores also increased more prominently in the intervention group than in the control group (*P* < 0.001). Short-term outcomes showed improvement in the intervention group compared to the control group (*P* < 0.05). Long-term outcomes revealed that the intervention group experienced better results than the control group in specialist visits, exercise stress tests, echocardiography, and readmissions (*P* < 0.05).

**Conclusion:**

Interventions such as improving the triage process and the PN are important in reducing disparities and improving patient outcomes. These findings underscore the effectiveness of tailored strategies in promoting equitable care in ED.

**Supplementary Information:**

The online version contains supplementary material available at 10.1186/s12939-025-02496-1.

## Background

Acute Coronary Syndrome (ACS) is the most common chronic progressive disease and a major public health challenge worldwide [1]. This disease is responsible for 1.7 million deaths annually, 80% of which occur in developing countries [2]. In Iran, this disease is responsible for approximately 4.6 deaths per 10,000 population [3]. Patients with ACS may present with symptoms such as sweating, shortness of breath, nausea and vomiting, and abdominal pain, but these symptoms are not always typical [4]. Evidence suggests that about 6% of people with ACS experience symptoms in the form of arrhythmias without pain [5].

In the emergency care of patients with ACS, the triage nurse is often the first provider to manage and identify patients’ clinical conditions requiring immediate attention; however, they often do not diagnose or prioritize ACS [5, 6]. Correct identification of patients, especially those with nonspecific symptoms, makes it difficult for the nurse to decide on triage level assignment or severity of illness [7]. Under-triage of patients with ACS and prolonged ischemic time is associated with adverse outcomes such as death and heart failure [8]. Inadequate staffing, cultural bias, language barriers, burnout, and stress among triage nurses contribute to poor triage and worsen patient safety [9]. Over-triage or under-triage also endangers equitable and timely access to care and the allocation of emergency resources; therefore, improving the timely assessment and management of ACS is a clinical priority in emergency care [10].

After proper triage, a patient with ACS requires prompt treatment and ongoing follow-up. However, the median time these patients receive and access emergency services varies from 30 min to 72 h [4]. Approximately 30 to 40% of patients with ACS do not receive timely treatment and coronary reperfusion [11]. A significant barrier to triage or emergency care of patients with ACS is healthcare disparity and the lack of uniform treatment for all patients [12]. Racism, gender, age, socioeconomic status, and lack of education and skills among healthcare providers are among the factors that contribute to disparities in the delivery of healthcare services to patients with ACS [13].

Disparities in care is a term used to describe the differences, variations, and inequalities in access to healthcare services [14]. Evidence suggests that disparities in access to healthcare services for patients with ACS are associated with poor outcomes. These outcomes may include increased patient mortality, length of stay and hospitalization, disease progression, self-reported outcomes, treatment costs, and decreased patient satisfaction with healthcare services [2]. Patients at risk for disparities are usually poorly identified and diagnosed in the Emergency Department (ED). Even over- and under-triage rates have been reported to be higher in EDs [15]. To reduce disparities and improve care for patients with ACS, in addition to improving the triage process, training healthcare workers in ethical principles, reviewing healthcare policies, increasing insurance coverage and telemedicine services, reducing prehospital delays, equipping ambulances with ECGs, and facilitating access to healthcare services can be mentioned [11, 15, 16].

Interventions to reduce disparities in the provision of emergency services to patients with ACS are mainly at the level of organizational and health system changes and sometimes require adequate funding and multi-sectoral coordination; therefore, there is a need for more practical, cost-effective, and effective interventions. Evidence suggests that the Emergency Severity Index (ESI) tool is used to assess all complaints and has significant limitations in accurately assessing cardiovascular risk factors and triaging patients with suspected ACS and predisposition to inequality [6]. A specialized triage tool such as HEART (history, electrocardiography, age, risk factors, troponin) score can lead to more accurate triage and improved health and disease outcomes in patients with ACS. It can also reduce the time to treatment, implement early therapeutic interventions, and eliminate unnecessary services [6]. Another essential intervention to reduce disparities in delivering quality services to patients with ACS is implementing a patient navigation (PN) program, which focuses primarily on vulnerable populations and aims to provide health care that reduces disparities. A PN is a support system or patient-centered care associated with reducing disparities and discrimination among patients, reducing hospital waiting times and length of stay, and improving quality of care [17]. In PN, a person with or without a healthcare background interacts with the patient individually, removing barriers to access, implementing recommended guidelines, and providing self-management and access to healthcare services for the patient [18]. One study found that PN in the ED can improve the quality of care. As many as 44% of staff in a survey agreed with the implementation of PN in the ED [19]. Therefore, given the importance of timely identification and management of patients at risk for disparities in the ED and the potential of the PN program to reduce disparities, the present study aimed to evaluate the impact of a disparities-reducing intervention on outcomes of patients with ACS in the ED.

## Methods

### Study design

This study is a randomized controlled clinical trial.

### Setting

The study setting is the ED of Imam Ali Hospital in Kermanshah, a specialized cardiac care center with four Coronary Care Units (CCU). The ED includes 14 emergency beds and eight bedside nurses per shift. On average, 180 to 220 patients with symptoms of ACS visit the department daily, amounting to approximately 72,000 patients annually. Upon arrival, nurses triage patients using the five-level ESI triage system. This study was conducted from October 2023 to February 2024.

### Participants

Inclusion criteria encompassed patients with confirmed or suspected ACS and lack of ST-elevation myocardial Infarction (STEMI) cases. Eligible participants had at least two socio-demographic vulnerabilities, such as advanced age, female gender, living in rural or underserved areas, low educational attainment, illiteracy, or a history of substance use. Further criteria required participants’ willingness to join the study, provision of informed consent, absence of psychiatric disorders, and 18 years or older. Patients included had no history of chest trauma, presented with typical or atypical chest pain or pressure associated with ACS, were admitted to the ED for a minimum of two hours, and were fully proficient in Persian. Chest pain onset had to be within the 12 h preceding arrival, and exclusion criteria covered pregnancy, acute infectious disease, or a history of previous myocardial infarction.

Patients were excluded from the study if they experienced mortality in the ED, were transferred to another healthcare facility, or discharged themselves against medical advice. Patients engaged in similar concurrent interventions, such as triage quality improvement programs or those with extensive cardiac injury necessitating urgent surgical intervention, were also excluded. Hemodynamically unstable patients requiring cardiopulmonary resuscitation upon arrival or those directly transferred from the ED to the CCU or catheterization lab were deemed ineligible.

### Sampling and sample size

The study population comprised cardiovascular patients presenting to the ED, with participants selected through convenience sampling from those diagnosed with ACS.

The following assumptions were used to determine the sample size: a significance level of α = 0.05, a statistical power of 80% (β = 0.20), and an effect size of 0.11. The expected incidence of cardiac events in the control group (P1 = 19%) was based on data reported by Frisch et al. [6]. For the intervention group, we anticipated a reduction in the incidence of cardiac events to 8% (P2), representing a clinically meaningful improvement. The effect size (0.11) was calculated based on the difference between these proportions and reflects a moderate impact of the intervention. We included a 10% anticipated dropout rate to account for participant attrition. Thus, although the initial calculation yielded a requirement of 120 participants per group, we increased the sample size to 132 per group to maintain statistical power, resulting in a total of 264 participants.

$$\:n=\frac{({z}_{1-\frac{\alpha\:}{2}}+{z}_{1-\beta\:}{)}^{2}}{{d}^{2}}({p}_{1}{q}_{1}+{p}_{2}{q}_{2})$$

### Randomization and blinding

The participants were randomly assigned to the study groups using a stratified block randomization method. Stratification was based on age (under 60 and 60 years or older) and gender (male and female) to ensure group balance. Considering the two strata, the sample allocation employed a 4-block randomization design, which resulted in four possible categories. The allocation sequence was generated by listing the block combinations (AABB, ABAB, ABBA, BBAA) and randomly selecting numbers between 1 and 6 using a random number table. The final treatment allocation list was based on the letter sequences AABB (1), ABAB (2), ABBA (3), BBAA (4), BABA (5), and BAAB (6).

An independent nurse coded the groups as A (intervention) and B (control), maintaining confidentiality until data analysis was completed. The data analyst, outcome assessor (data collector nurse), and study participants were all blinded to the group allocation. However, blinding the interventionist was not feasible due to the nature of the study.

### Data collection tools

The data collection tools included a demographic information form, a Numerical Rating Scale (NRS), a patient opinion of pain management questionnaire, a threat perception scale, an illness perception scale, and short—and long-term outcomes questionnaires.

#### Demographic information

The demographic information form was completed at the beginning of the study through patient interviews and medical records. It included details such as gender, age, education level, marital status, employment status, body mass index (BMI), place of residence, and type of health insurance coverage.

#### Pain intensity

The intensity of pain experienced by using the NRS. This scale ranges from 0 to 10, where patients select the number that most accurately reflects their pain level. A score of zero indicates the absence of pain, while a score of ten represents the worst possible pain. In the evaluation of chest pain, scores of 1 to 3 were categorized as mild pain, 4 to 6 as moderate pain, and 7 to 10 as severe pain [20]. This scale was completed through self-reporting at admission and discharge from the ED.

#### Patient opinion of pain management

The Patient Opinion of Pain Management tool has a 12-item adapted from the American Pain Society’s Patient Outcome Questionnaire and the Patient Opinion of Pain Management Tool. This questionnaire consists of various sections, with items 1–4 addressing personal information, items 5–7 focusing on different aspects of pain intensity, and items 8–12 evaluating satisfaction with pain management. For this study, only items 8–12 will be utilized to assess patient satisfaction with pain management provided by nursing staff. Scoring for the questions is based on a Likert scale ranging from 1 to 6, where one indicates “very dissatisfied” and six means “very satisfied.” The minimum possible score is 5, while the maximum score is 30. Higher scores reflect greater satisfaction with nursing services in pain management. Sepahvand et al.‘s study confirmed the content validity of this tool, and its reliability was established using the test-retest method, yielding a correlation coefficient of *r* = 0.86 [21]. This tool was completed through self-reporting at discharge from the ED.

A standardized pain management protocol was applied uniformly across the intervention and control groups. This protocol included the administration of opioid and non-opioid analgesics based on patient-reported pain levels and clinical judgment, as well as supportive non-pharmacological strategies, such as massage therapy, as per the hospital’s routine practice.

#### Threat perception

This scale consists of six questions, each rated on a four-point Likert scale: (1) “does not apply to me at all,” (2) “slightly applies to me,” (3) “moderately applies to me,” and (4) “completely applies to me.” The minimum possible score on this questionnaire is 6, while the maximum score is 24. A higher score indicates a more excellent perception of threat. The reliability of this scale was examined by Zhu et al., who reported a Cronbach’s alpha of 0.79, indicating acceptable internal consistencyally [22]; in the study by Veiskramian et al., the content validity of this questionnaire was confirmed for these patients, and the reliability of the scale was determined through test-retest methodology, yielding a correlation coefficient of 76% [23]. This scale was completed through self-reporting at the time of admission and discharge from the ED.

#### Illness perception

This questionnaire is a concise 9-item, designed to evaluate both the emotional and cognitive representations of illness. The questions assess various dimensions, including consequences, duration, personal control, treatment control, identity, concern, illness comprehension, emotional response, and perceived causes of the illness. The scoring range for the first eight items is from one to ten, while the ninth question is an open-ended item that asks respondents to identify the three main reasons for their illness in order of significance. The minimum score on this questionnaire is zero, and the maximum score is 80. Taghizadeh et al. confirmed this questionnaire’s reliability, with Cronbach’s alpha reported between 76% and 82% in the study [24]. This questionnaire was completed through self-reporting at admission and discharge from the ED.

#### Short-term outcomes

This questionnaire records vital time intervals, including door-to-ECG, door-to-physician, door-to-painkiller time, and ED stay. It also records the frequency of cardiac interventions, including the number of catheterizations, angiographies, and cardiac surgeries performed. This questionnaire was completed using the medical records at admission and discharge from the ED.

#### Long-term outcomes

Major cardiac events include a number of major cardiac events, recurrent myocardial infarction during hospitalization, and cardiac interventions. Access to care is assessed based on the number of follow-up visits to a cardiologist and the frequency of exercise stress tests, echocardiograms, and readmission conducted within one month after discharge. This information was obtained via telephone.

### Interventions

**Fig. 1:**
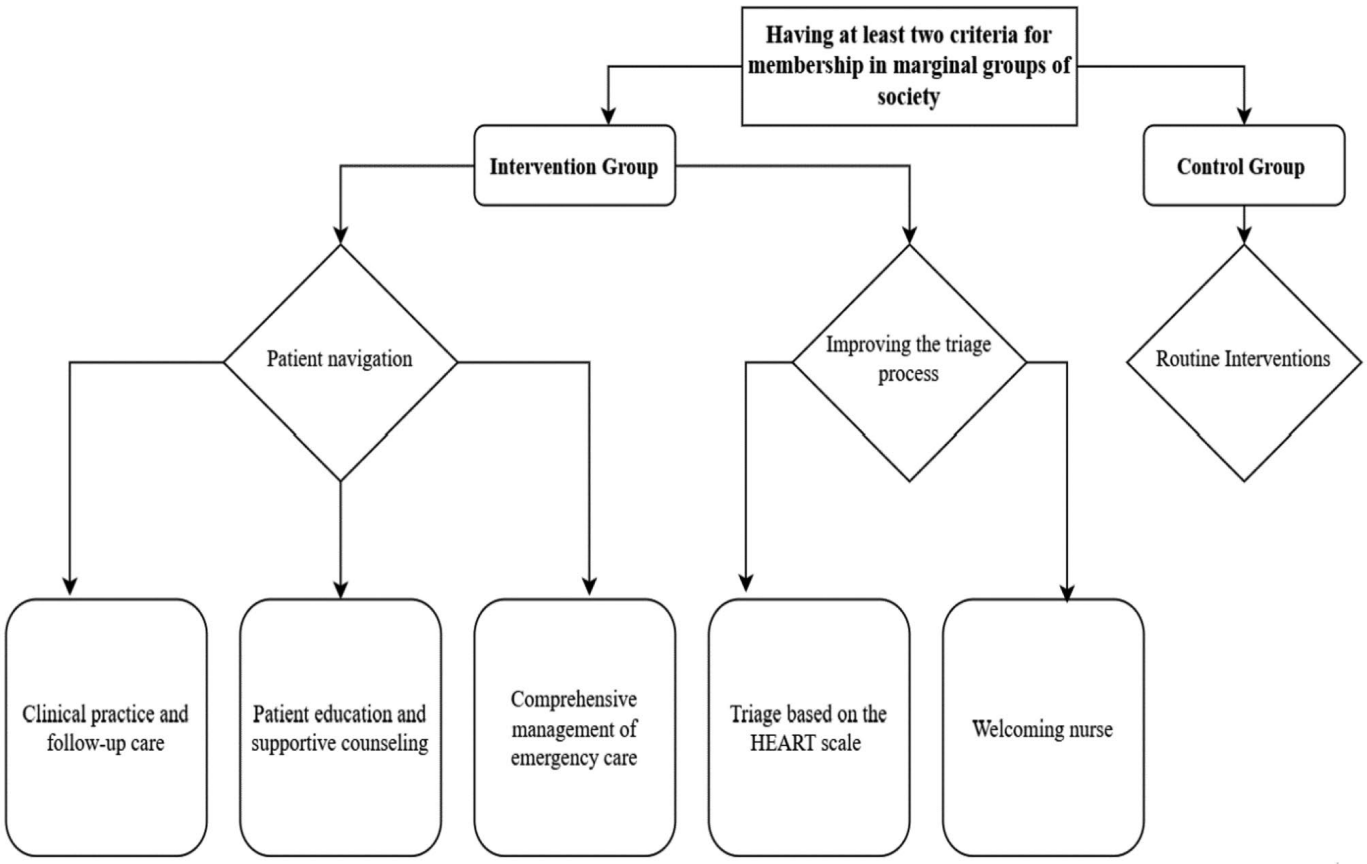
Diagram of the intervention

One week before the intervention, the lead researcher conducted three group training sessions for ED staff to ensure their cooperation and familiarity with the intervention. Routine procedures were conducted in the control group. For the intervention group, the program was implemented with two main components: “triage process improvement” and “patient navigation program” (Fig. 1).

#### Triage process improvement

Upon arrival in the ED, patients in the intervention group underwent the improved triage process, which included:

##### Welcoming nurse

Upon the patient’s arrival at the triage unit in the ED and determination of the triage level using the ESI, an experienced nurse specializing in cardiac triage they have assumed the role of a welcoming nurse. The responsibilities of this nurse in the current intervention were as follows: The nurse first introduced themselves to the patient and their family, offering a warm greeting and engaging in a brief conversation. Actions by this nurse included obtaining the patient’s medical history focused on typical and atypical symptoms of ACS, initiating cardiac monitoring, sending specific blood tests to diagnose ACS, recording a 12-lead electrocardiogram (ECG), and interpreting it in ten minutes, assessing the severity of chest pain using the NRS, and administering analgesics based on the pain score. Subsequently, the nurse assistant with the wheelchair transferred the patient to the triage unit’s observation beds, and the welcoming nurse’s contact information was provided to the patient and their family. Special attention was given to maintaining patient privacy in the triage unit.

##### Patient assessment using the HEART score

The second phase of the triage improvement process involved risk stratification of patients based on the HEART score conducted by the welcoming nurse. The HEART score is a tool designed to predict the likelihood of ischemic events in patients presenting to the ED with chest pain. Its accuracy, speed, and reliable outcomes facilitated the safe discharge of low-risk patients and the preparation of higher-risk patients for invasive interventions.

Using the HEART score, five parameters were evaluated: History, ECG, Age, Risk factors, and Troponin levels. Each parameter was scored on a scale of 0 to 2, where highly suspicious findings were assigned a score of 2, moderately suspicious findings received a score of 1, and completely nonspecific findings were scored 0 [25]. Poldervaart et al. developed this tool, and its validity and reliability have been evaluated [26]. (Supplementary File 1).

#### Patient navigation program

This study’s Patient Navigation (PN) program was designed to support patients diagnosed with ACS during the critical transition from emergency care to post-discharge follow-up. The intervention involved structured follow-up, patient education, and care coordination.

The navigator was an experienced nurse with over ten years of clinical experience in cardiovascular care and underwent a dedicated 20-hour training program specifically designed for this study. The training covered communication skills, health coaching, discharge planning, and knowledge of local health and social support resources.

To ensure cultural appropriateness and responsiveness to the socio-demographic characteristics of the study population—including lower income, limited formal education, and high prevalence of comorbidities—the PN program incorporated simplified educational materials, visual aids, and individualized counseling based on each patient’s literacy level and family context. The navigator also conducted home follow-ups via phone to accommodate transportation or access barriers, and referrals to social services were made when needed.

The navigator maintained regular contact with participants over one month, assisting with medication adherence, follow-up appointments, and lifestyle modifications. Her efforts aimed to reduce gaps in care and improve short-term outcomes following discharge. This program was implemented by the nurse in the following three areas:

##### Supportive education and counseling

In this activity, the navigator nurse spent 20 min in a private setting using effective communication techniques (e.g., active listening, reflection, and unconditional acceptance of the patient) and interviews to identify the patient’s emotional, psychosocial, and social needs, as well as barriers to receiving care. The patient was encouraged to express concerns, mental challenges, feelings of sadness, anxiety, and stress. Reassurance was provided to the patient and their family that the navigator nurse would remain alongside them throughout the care and treatment process, liaising with other healthcare providers and hospital departments.

Additionally, face-to-face educational sessions lasting 10–15 min were conducted using appropriate visual aids tailored to the patient’s and caregivers’ language and literacy levels. These sessions covered topics related to the disease and its management. Education was provided when the patient was in a stable physical and mental condition, particularly during hemodynamic stability. Detailed educational content is presented in Supplementary File 2.

##### Emergency care comprehensive’s management

While overseeing the patient’s diagnostic and treatment plans and identifying flaws and critical areas in emergency services (such as increased waiting times), the navigator nurse worked to expedite the referral and transfer of the patient from the ED to other departments. This was achieved by fostering collaboration with physicians and other ED staff, ensuring the coordination of patient transfer to other departments. The patient’s environment was assessed for adherence to safety principles and potential fall hazards, with essential corrective actions to mitigate risks. At this stage, the navigator also evaluated the patient’s socioeconomic status by reviewing monthly income, employment status, housing, and insurance coverage. If necessary, patients were referred to social work for further assistance.

##### Clinical action and follow-up care

This section focused on clinical status monitoring, comorbidity screening, medication management, and follow-up care.

During the patient’s stay in the ED, the navigator nurse, in collaboration with the emergency nursing staff, enhanced clinical monitoring of the patient’s hemodynamic status and ensured that the patient received all necessary therapeutic and pharmacological interventions.

Comorbidities were assessed using the Charlson Comorbidity Index (CCI), a validated and reliable scale of 19 items evaluating the presence of comorbid conditions. Each condition was assigned a score of 1, 2, 3, or 6 [27]. The CCI results were subsequently shared with the emergency physician and the patient’s attending physician.

To support medication management, a disease-specific medication booklet was provided to patients in collaboration with a clinical pharmacist. Additionally, elderly patients received medication reminder boxes to improve adherence.

During discharge, the navigator prepared a detailed follow-up care plan, including the schedule for medical tests, echocardiography, and clinic visits or follow-up appointments. This plan was handed over to the patient or their primary caregiver.

Post-discharge, the navigator maintained contact with the patient or their primary caregiver for three weeks, making three phone calls per week—these calls aimed to monitor the success or challenges in referrals and continuity of care. The navigator also provided reminders about self-care practices, attending follow-up visits, and completing future diagnostic and therapeutic interventions.

### Fidelity of the study

To ensure the quality of the study, the researcher developed a service delivery checklist for patient care. This checklist documented various elements, including the time or duration of each interaction, appointments, patient accompaniment, the number and duration of phone calls, the frequency of referrals to other healthcare departments or physicians, as well as all interactions and barriers related to transportation, social support, care follow-up, financial issues, health literacy/education, and language or communication challenges with the patient.

Two nurses employed in the study setting performed the role of welcoming nurse, while the principal investigator performed the role of navigator nurse. The welcoming nurses, triage nurses, and navigator nurses all had at least five years of experience caring for cardiac patients and managing emergencies for patients with ACS. They also received specialized training in therapeutic communication skills, working with diverse populations, and coordinating and overseeing emergency services.

Data was collected by an independent evaluator who was not involved in the navigation intervention. This included completing the questionnaire, collecting individual and clinical information forms, and recording the primary study outcomes. Data collection was managed by a trained nurse independent of the intervention team to maintain objectivity and minimize bias.

### Data analysis

Data were analyzed using SPSS software (v.24). Proportions, central tendency measures, and appropriate dispersion indices were reported. The normality of continuous variables was assessed using the Kolmogorov-Smirnov test and graphical methods. If the data followed a normal distribution, an independent t-test was used to compare means or mean changes between the two groups, and a paired t-test was applied to compare pre-and post-intervention means. For non-normally distributed data, suitable non-parametric tests were utilized. Additionally, the Chi-square test was employed to compare categorical variables between groups. When appropriate conditions were met, covariance analysis (ANCOVA) or the general linear model (GLM) was used for data analysis. Potential confounding variables were included as covariates in the analysis. Mean imputation was applied to address missing data and minimize participant exclusion. Results were reported at a significance level of 0.05.

## Results

**Fig. 2:**
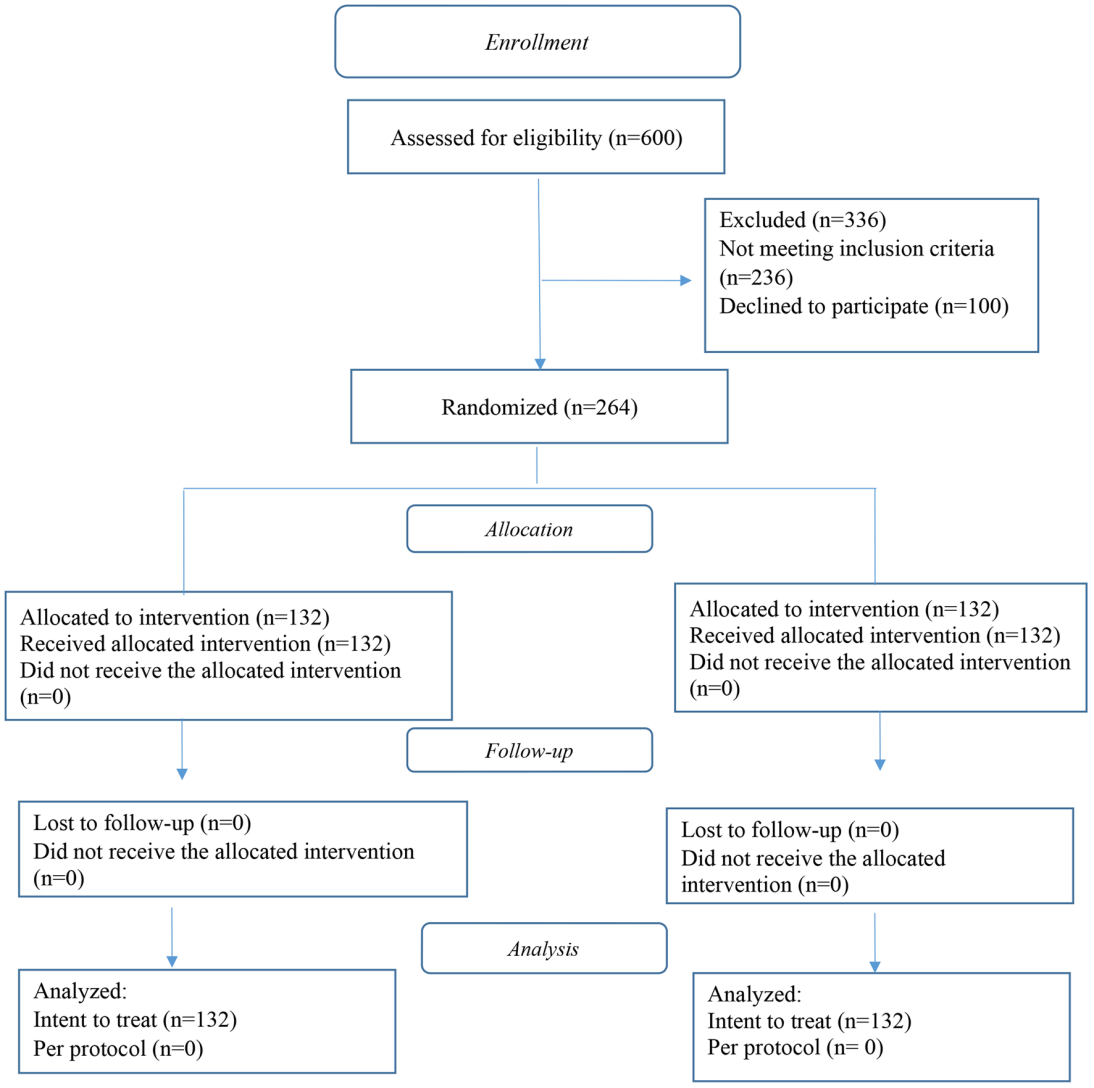
Consort diagram

Six hundred patients with ACS were assessed for eligibility to participate in the study, and ultimately, 264 patients were enrolled (Fig. 2). The results showed no statistically significant difference in demographic and basic information between the two groups (*P* > 0.05) (Table 1). The Chi^2^ test showed no difference in type of insurance (*P* = 0.77).

**Table 1:** Comparison of demographic and baseline information across study groups

Variables	Intervention *N* (%)	Control *N* (%)	Chi²	*P*-value
**Gender**			0.15	0.99
Male	67 (50.8%)	66 (50%)		
Female	65 (49.2%)	66 (50%)		
**Age**			0.243	0.711
≤ 60 years	72 (54.5%)	68 (51.5%)		
> 60 years	60 (45.5%)	64 (48.5%)		
**Education**			1.962	0.11
Less than middle school	121 (51.5%)	114 (48.5%)		
Middle school to below high school	9 (39.1%)	14 (60.9%)		
High school or above	2 (33.3%)	4 (66.7%)		
**Marital Status**			1.264	0.532
Married	99 (75%)	94 (71.2%)		
Single	32 (24.2%)	35 (26.5%)		
Other	1 (0.8%)	3 (2.3%)		
**Employment Status**			7.858	0.249
Self-employed	17 (12.9%)	7 (5.3%)		
Employee	3 (2.3%)	1 (0.8%)		
Housewife	56 (42.4%)	52 (39.4%)		
Unemployed	14 (10.6%)	20 (15.2%)		
Retired	6 (4.5%)	9 (6.8%)		
Laborer	18 (13.6%)	19 (14.4%)		
Farmer	18 (13.6%)	24 (18.2%)		
**BMI**			3.991	0.136
< 18.5	3 (2.3%)	3 (2.3%)		
18.5–24.9	89 (67.4%)	103 (78%)		
> 25	40 (30.3%)	26 (19.7%)		
**Residence**			2.576	0.462
City	6 (4.5%)	6 (4.5%)		
Suburban	48 (36.4%)	53 (40.2%)		
Rural	78 (59.1%)	71 (53.8%)		
Homeless	0 (0%)	2 (1.5%)		
**History of Chronic Disease**			1.598	0.255
No	46 (34.8%)	56 (42.4%)		
Yes	86 (65.2%)	76 (57.6%)		

The opinion of pain management—measured only post-intervention—was significantly higher in the intervention group (22.21 ± 3.64) than in the control group (17.14 ± 3.82) (*P* < 0.001). The results also showed that after the intervention, pain intensity and threat perception decreased more significantly in the intervention group compared to the control group (*P* < 0.001). Furthermore, illness perception scores increased more prominently in the intervention group than in the control group (*P* < 0.001) (Table 2).

**Table 2:** Comparison of pain intensity, threat perception, and illness perception between study groups

Variable	Group	Mean ± SD (Before)	Mean ± SD (After)	Change %	Point Estimate	*P*-value ^a^	*P*-value ^b^
**Pain Intensity**	Intervention	6.59 ± 2.17	3.50 ± 1.52	-46.89%	1.257	< 0.001^c^	< 0.001^d^
	Control	6.08 ± 2.01	4.37 ± 1.94	-28.13%	0.910	< 0.001^c^	
**Threat Perception**	Intervention	16.46 ± 3.90	11.54 ± 3.62	-29.9%	0.975	< 0.001^c^	< 0.001^d^
	Control	15.88 ± 3.89	15.28 ± 4.07	-3.78%	0.263	0.003^c^	
**Illness Perception**	Intervention	49.34 ± 8.84	61.09 ± 7.89	-19.23%	1.509	< 0.001^c^	< 0.001^e^
	Control	50.56 ± 8.93	52.0 ± 7.71	-2.77%	0.276	0.002^c^	

a Within group

b Between groups

c Paired t-test

d Independent t-test

e ANCOVA

The Chi² test revealed that a significantly higher proportion of patients in the intervention group received painkillers in the ED compared to the control group (*P* = 0.03). Other short-term outcomes also showed improvement in the intervention group compared to the control group (*P* < 0.001) (Table 3).

**Table 3:** Comparison of short-term outcomes between study groups

Variables	Intervention (Mean ± SD)	Control (Mean ± SD)	t	*P*-value
Door-to-ECG ^a^	5.65 ± 1.39	7.47 ± 2.67	-6.95	< 0.001
Door-to-physician ^a^	7.28 ± 1.67	9.29 ± 2.89	-6.91	< 0.001
Door-to-pain killer ^a^	16.01 ± 7.83	22.29 ± 10.61	-4.64	< 0.001
ED stay ^b^	186.81 ± 51.34	224.50 ± 54.29	-5.79	< 0.001
Waiting time ^b^	0.32 ± 0.59	1.60 ± 1.30	-10.27	< 0.001

a Minutes

b Hours

The findings indicated that one month after discharge, the intervention group had a higher frequency of specialist visits, exercise stress tests, and echocardiography compared to the control group (*P* < 0.001), while the rate of readmission was lower (*P* = 0.02). No statistically significant difference was observed in a number of major cardiac events, recurrent myocardial infarction during hospitalization, and cardiac interventions in the ED (Table 4).

**Table 4:** Comparison of long-term outcomes between study groups

Variables	Intervention *N* (%)	Control *N* (%)	Statistic	*P*-value
**Number of major cardiac events**			Chi² = 0.47	0.6
Once	91 (90.1%)	79 (92.9%)		
Twice	10 (9.9%)	6 (7.1%)		
**Follow-up visits**			z = 7.34	< 0.001
None	2 (1.5%)	33 (25%)		
Once	86 (65.2%)	92 (69.7%)		
Twice	44 (33.3%)	7 (5.3%)		
**Exercise stress tests**			z = 6.15	< 0.001
None	46 (35.1%)	96 (72.7%)		
Once	83 (63.4%)	36 (27.3%)		
Twice	2 (1.5%)	0 (0%)		
**Echocardiography**			z = 7.37	< 0.001
None	1 (0.8%)	33 (25%)		
Once	100 (76.3%)	97 (73.5%)		
Twice	30 (22.9%)	2 (1.5%)		
**Readmissions**			Chi² = 5.49	0.02
No	71 (53.8%)	52 (39.4%)		
Yes	61 (46.2%)	80 (60.6%)		
**Recurrent myocardial infarction during hospitalization**			Chi² = 1.15	0.38
No	122 (93.1%)	118 (89.4%)		
Yes	9 (6.9%)	14 (10.6%)		
**Cardiac interventions**			Chi² = 0.00	0.67
No	2 (9.1%)	2 (8.7%)		
Yes	20 (90.9%)	21 (91.3%)		

## Discussion

The current study showed that interventions such as improving the triage process and the PN are important in reducing disparities and improving patient outcomes. The findings show that threat perception decreased in the intervention group, which is consistent with the study by Alikhah et al. [28]. An intervention to reduce disparity and create patient safety can reduce threat perception. It can be said that PN, face-to-face education about the disease, and, most importantly, patient-centered care in this intervention increase patient’s awareness and reduces their anxiety and sense of danger. In the study by Hamedy Soliman et al., it was reported that anxiety symptoms were reduced by patient-centered care, comprehensive nursing attention to patient’s psychological needs, and massage therapy [29]. However, in the study by Soleimani et al., a collaborative care program was used to manage patients’ stress and feelings of danger. The results showed that increasing family presence alone did not affect patients’ anxiety [30]. It can be said that managing anxiety and reducing feelings of danger in patients cannot be achieved without comprehensive support, education, and increasing patient awareness.

The current findings indicate that the opinion of pain management was significantly more favorable in the intervention group, likely due to the integrated approach of specialized triage and comprehensive PN. Although a standardized pain management protocol—including both opioid and non-opioid analgesics and non-pharmacological strategies such as massage—was applied equally in both groups, the intervention group benefited from additional support through the PN program, which involved tailored follow-up and personalized education. These elements likely contributed to greater patient satisfaction and perception of effective pain management. Pain intensity also decreased more significantly in the intervention group. Pain considerably impacts patients’ quality of life and clinical outcomes. In patients with ACS, unmanaged pain can trigger systemic cardiovascular responses, such as elevated blood pressure, increased heart rate, and emotional distress. Given its connection with anxiety and emotional burden, effective pain control is crucial in this population [31]. Evidence shows that pain management techniques are very important because they provide optimal pain relief, increase patient satisfaction, and improve overall outcomes [32]. In general, a patient-centered pain management program that includes non-opioid analgesics, regional anesthesia, and careful selection of opioid medications can result in adequate analgesia and satisfaction with care [33]. In the study by Zahid et al., in addition to responsible use of medications using effective patient-centered tactics, individual needs were addressed, communication was improved, and resources were allocated for multifaceted pain management in patients, resulting in increased satisfaction with pain management in cardiac patients [32]. In another study, specific interventions such as motivational interviewing, education, shared decision-making, rapid assessment and risk stratification, and management of acute chest pain achieved high levels of satisfaction, pain management, and minimal uncertainty in patients with ACS. As a result, it can be said that flexible and patient-centered care leads to greater patient satisfaction with pain management [34], consistent with this study’s findings. Patient-centered care and attention to patients’ needs can reduce pain and increase patients’ satisfaction with pain management. As a result, these interventions create equal opportunities for understanding the disease and making informed choices, increase patients’ trust in the health system, and reduce feelings of discrimination among them.

This study also showed that improving the triage process can also be effective in managing patient anxiety and pain. In the study by Sharp et al., it was reported that using the improved triage process to reduce waiting time and provide a safe environment and special attention to the patient can improve patient pain management [35], which is consistent with the findings of this study. It can be concluded that in addition to the importance of pain control in patients with ACS, this issue requires a multifaceted approach that is achieved together with the use of appropriate analgesics, flexible and patient-centered care, proper communication, and attention to the specific needs and preferences of the patient. In general, paying attention to the specific needs of patients through personalizing healthcare services and providing targeted care can reduce inequality.

Another notable finding of this study is that supportive education and counseling on topics such as ACS, increasing understanding and knowledge of the disease, and identifying the patient’s emotional and psychological needs increased disease perception of the intervention group compared to the control group. The study by Weibel et al. reported that education, identification of emotional and psychosocial needs, and the presence of a caregiver (navigator) as the patient’s therapeutic interface improved patient awareness, knowledge, and, subsequently, perception of the disease [36]. The results of a clinical trial to reduce risk and increase disease understanding in patients with ACS showed that a mindfulness-based intervention can reduce negative affect (stress) and risk perception and increase the person’s perception or awareness of their disease and condition [37], which is consistent with the findings of this study. Increased perceptions of disease control may increase treatment adherence [38]. More accurate knowledge of cardiovascular risk factors may contribute to better decision-making in patients with ACS by accurately assessing the perceived risk of a cardiac event and reducing unrealistically optimistic perceptions in these patients [39]. Supportive education and counseling for patients with social and cultural inequalities leads to equal opportunities to receive medical services and make more informed choices.

Our findings show that improved triage processes and PN can reduce wait times, length of stay, and service delivery time in the ED. Given the importance of time in treating ACS and the critical condition of these patients, it is possible to reduce wait times and expedite diagnostic and therapeutic interventions for these patients by modifying and improving the triage process. In addition, with a comprehensive support program and the PN, quality services are provided to patients, and the length of hospital stay is reduced. As a result, reducing waiting times and lengths of stay in the ED can reduce inequality by improving equitable access to health services. Consistent with the results of this study, Karla Andrade et al. reported that rapid and appropriate management of patients with ACS can reduce the waiting time to obtain an ECG and mortality [40]. Another study reported that the implementation of cardiac triage positively impacted time-based triage indicators for patients in the ED [41].

Current evidence suggests that PN significantly reduces readmissions by providing a supportive system, reducing barriers to care, and improving outcomes. Wu et al. reported that using a quality improvement plan improved several indicators of the evidence-based care process; however, no changes in the reduction of major cardiac events were reported [42]. The study by Kalter-Leibovici (2017) showed that mortality and readmission rates decreased after the intervention, indicating improved access to care for these patients. In addition, the psychosocial support provided by nurses led to improved patient satisfaction with treatment, decreased depression, and increased daily walking [43]. The disparity reduction intervention and PN can improve access to care and reduce readmission rates in patients with ACS.

The findings indicate that the intervention group had a higher frequency of cardiac rehabilitation one month after discharge, which is consistent with the results of previous studies [17, 44]. A possible explanation could be that the PN made patients more aware of the need to use these services. Increasing patients’ awareness of their needs and receiving timely medical services can reduce gaps between patient groups and create better treatment outcomes for them. In fact, PN prepares patients for upcoming visits and encourages them to visit the hospital before pain and illness worsen [44, 45].

While the intervention demonstrated positive outcomes in this study, several challenges were encountered during its implementation, which could be valuable considerations for replication in other clinical settings. One of the main difficulties involved coordinating the triage process improvements, especially within the time constraints of the ED. The role of the specialized triage nurse—who single-handedly handled patient assessment, immediate interventions, and privacy maintenance—required careful planning and time management.

Additionally, the complexity of addressing patients’ sociodemographic and cultural needs in the PN program posed a challenge. The navigator’s ability to adapt educational content to diverse literacy levels and provide personalized care in a busy clinical environment was crucial but demanding.

Moreover, although integrating the HEART score as a tool for risk stratification was beneficial, it required focused training and consistent application by the designated triage nurse. Ensuring effective communication between the navigator, ED staff, and other healthcare professionals was also critical to avoid delays in patient care and referrals.

Notwithstanding important findings, this study had limitations that need to be considered. First, it was conducted only in the ED of a single hospital in Iran, which may limit the generalizability of the findings to other hospitals, regions, or healthcare systems with different structures and resources. Future research should replicate this study in varied clinical settings and countries to validate the results across diverse cultural and healthcare contexts.

Second, the use of questionnaires in this study may be affected by response bias and individual differences in the interpretation of questions.

Third, blinding the interventionist was not feasible due to the nature of the intervention. However, a more rigorous approach could have been implemented to blind the outcome assessors. For instance, having a separate team uninvolved in the intervention process to collect and evaluate outcome data could have helped reduce detection bias. The lack of proper blinding at this stage introduces a risk of subjective influence on the outcome assessment and should be acknowledged as a methodological limitation.

Fourth, in this study, the long-term outcomes are measured only up to one month after discharge. Given the chronic nature of ACS, longer follow-up periods (e.g., 6 months or 1 year) would provide more insight into the sustained impact of the intervention.

Fifth, as the study was conducted in Iran, the findings may be influenced by specific cultural norms, healthcare policies, and delivery practices that differ from those in other countries. These contextual differences can impact patient engagement, intervention efficacy, and measurement tools, thereby limiting broader applicability. For instance, family involvement in care decisions, respect for healthcare authority, and communication preferences may shape how patients receive and respond to navigation services. Additionally, differences in access to post-discharge care and social support systems could influence intervention outcomes in other settings.

Despite these cultural and systemic particularities, the core components of the PN program—such as structured follow-up, patient education, and care coordination—are conceptually transferable. However, adaptation to local languages, beliefs, and healthcare infrastructures would be essential for effective implementation elsewhere. Future studies should explore the cultural tailoring of similar interventions to assess feasibility and effectiveness in different contexts.

To improve future studies, it is recommended to extend the follow-up period to evaluate the long-term impact of the intervention on patient mortality, readmission rates, and quality of life. Additionally, broadening the inclusion criteria to encompass a wider range of patients could enhance the generalizability of the findings. Incorporating a qualitative component, such as interviews with patients and healthcare providers, would offer deeper insights into the barriers and facilitators of the intervention. Finally, conducting a cost-effectiveness analysis is essential to determine whether the benefits of the intervention justify its costs and ensure resources are utilized optimally.

## Conclusion

The present study shows that interventions to reduce disparities can improve ACS patients’ satisfaction with care, reduce inequalities, and pay more attention to vulnerable and disadvantaged groups in society. Implementing such interventions with PN can improve pain management and access to health services, increase patient awareness of their disease, reduce feelings of danger and anxiety, and lead to early identification of ACS patients and reduced waiting times. Such patient-centered interventions, combined with an improved triage process, can reduce the rate of major cardiac events and mortality in the ED by reducing waiting times for medical services such as ECGs, physician visits, and hospitalization.

## Electronic supplementary material

Below is the link to the electronic supplementary material.

Supplementary Material 1

Supplementary Material 2

## Data Availability

The datasets generated and analyzed in this study are not publicly available because they contain individual informants, but they are available from the corresponding author upon reasonable request.

## Abbreviations

ACS: Acute Coronary Syndrome
PN: Patient Navigation
ED: Emergency Department
CCI: Charlson Comorbidity Index
