# Supplementary material for: The impact of a disparity-reduction intervention on outcomes of patients with acute coronary syndrome in the emergency department: a clinical trial
Source: Int J Equity Health. 2025 May 12;24:133. doi: 10.1186/s12939-025-02496-1 (PMC12067677; doi:10.1186/s12939-025-02496-1)
Supplement: Supplementary file 1 — Supplementary Material 1 [file 12939_2025_2496_MOESM1_ESM.docx]

**Supplementary File 1. Risk Stratification Based on the HEART Score**

| **Score** | **0** | **1** | **2** |
| --- | --- | --- | --- |
| **History** | No risk | Moderate-risk history | High-risk history |
| **ECG** | Normal | Minor abnormality | Significant ECG abnormality |
| **Age** | Below 45 years | 45 to 65 years | 65 years and older |
| **Risk factors** | No risk factors | 1 to 2 risk factors | More than three risk factors |
| **Troponin** | Within normal limits | 1 to 2 times the standard limit | 3 times the standard limit |
