# Supplementary material for: The impact of a disparity-reduction intervention on outcomes of patients with acute coronary syndrome in the emergency department: a clinical trial
Source: Int J Equity Health. 2025 May 12;24:133. doi: 10.1186/s12939-025-02496-1 (PMC12067677; doi:10.1186/s12939-025-02496-1)
Supplement: Supplementary file 2 — Supplementary Material 2 [file 12939_2025_2496_MOESM2_ESM.docx]

**Supplementary File 2. Patient Education Content during the Intervention**

| **Education Topic** | **Details** |
| --- | --- |
| **Warning Signs of ACS** | Chest pain, shortness of breath, low blood pressure, loss of consciousness, sweating, chest tightness, etc., are explained, emphasizing the differences in symptoms between men and women. Patients are advised to call emergency services in case of shortness of breath or rapid breathing. If experiencing weight gain or ankle swelling, they should consult a doctor. A drop in blood pressure or loss of consciousness may indicate cardiac arrhythmia. Heart attacks can sometimes occur without pain and may present as indigestion, sweating, or fatigue. Cardiac pain may manifest as a pressing sensation or sharp pain between the shoulder blades or radiate to the arm and jaw. |
| **Illness Perception and ACS Risk Factors** | The nature, duration, and controllability of the disease and risk factors such as unhealthy lifestyles, specific social and individual habits, gender, smoking, high blood pressure and cholesterol levels, physical inactivity, obesity, family history, stress, etc., are explained. |
| **Self-Care in ACS** | Education includes quitting smoking, consuming fish at least twice a week, engaging in daily physical activity for at least 30 minutes or 75-150 minutes weekly, eating high-fiber foods, limiting salt, fat, and carbohydrate intake, accessing cardiac care services, adhering to medication regimens, the importance of regular cholesterol and blood sugar evaluations, follow-up physician visits, managing stress, and how to contact emergency services. |
